# Community-Based Rehabilitation and Patient-Centered Outcomes in Survivors of Critical COVID-19 Attending an Intensive Care Recovery Clinic

**DOI:** 10.1016/j.arrct.2025.100484

**Published:** 2025-06-15

**Authors:** Felipe González-Seguel, Evan Haezebrouck, Lindsey E. Fresenko, Carla M. Sevin, Stacey Slone, Ashley Montgomery-Yates, Anna G. Kalema, Lori Ginoza, Clarisa Martinez, Michelle Biehl, Soibhan R. Kelley, Joshua K. Johnson, Matthew F. Mart, Kirby P. Mayer

**Affiliations:** a Department of Physical Therapy, College of Health Sciences, University of Kentucky, Lexington, KY; b School of Physical Therapy, Clínica Alemana Universidad del Desarrollo, Chile; c Department of Rehabilitation Services, University of Michigan Health, Ann Arbor, MI; d College of Health and Human Services, School of Exercise and Rehabilitation Sciences, University of Toledo, Toledo, OH; e Division of Allergy, Pulmonary, and Critical Care, Vanderbilt University Medical Center, Nashville, TN; f Dr. Bing Zhang Department of Statistics, University of Kentucky, Lexington, KY; g Internal Medicine, College of Medicine, University of Kentucky, Lexington, KY; h Division of Biokinesiology and Physical Therapy, University of Southern California, Los Angeles, CA; i Department of Physical Medicine and Rehabilitation, Cleveland Clinic, Cleveland, OH; j Division of Physical Therapy, School of Medicine, Duke University, Durham, NC; k Geriatric Research, Education, and Clinical Center (GRECC), Tennessee Valley Veterans Affairs Healthcare System, Nashville, TN

**Keywords:** COVID-19, Post intensive care syndrome, Critical illness, Physical therapy, Occupational therapy, Patient-centered outcomes

## Abstract

**Objective:**

To examine the occurrence of physical and cognitive impairments among survivors of critical coronavirus disease of 2019 (COVID-19) who attend an intensive care unit (ICU) recovery clinic and describe their utilization of community-based rehabilitation (physical and occupational therapy).

**Design:**

Retrospective, observational cohort study and multisite practice analysis.

**Setting:**

ICU recovery clinics at 4 academic medical centers.

**Participants:**

Adults (median age 56 [interquartile range, {IQR}, 47–64] years, 60% female) surviving acute respiratory failure caused by COVID-19 who required advanced respiratory support.

**Main outcome measures:**

Six-minute walk test (6MWT) and Montreal Cognitive Assessment (MoCA).

**Results:**

Patients attended the ICU recovery clinic (n=163) in a median of 43 (IQR, 30-60) days after discharge. Ninety-four patients (58%) participated in at least 1 community-based rehabilitation session, 52 (32%) never participated, and 17 (10%) did not have data available. Patients walked a median [IQR] of 282 [150-390] meters on the 6MWT, and the median Short Physical Performance Battery (SPPB) score was 8 [4-11] with 63% of patients classified as physically frail (score ≤9/12). The median MoCA score was 26 [22-27], with 37% at least mild cognitive impairment (score of ≤26). Among patients who were driving before ICU admission, 44% had not returned to driving after hospitalization, and an additional 21% reported driving with new limitations. Patients who participated in at least 1 community-based rehabilitation session had longer ICU lengths of stay as well as worse performance on the 6MWT and SPPB at discharge compared with individuals not receiving postdischarge rehabilitation (*P*<.001).

**Conclusions:**

Survivors of acute respiratory failure caused by critical COVID-19 who attended an ICU recovery clinic are at high risk of physical and/or cognitive impairments. Two-thirds of survivors participated in physical or occupational therapy at home or an outpatient center after hospital discharge. Patients with longer lengths of stay and more physical impairments at discharge are more likely to participate in community-based rehabilitation interventions.

Survivors of critical illness are at risk of developing new or worsening physical,^1^, ^2^, ^3^ mental,^4^^,^^5^ or cognitive^6^ impairments known as postintensive care syndrome (PICS).^7^ Despite recognition of PICS and a growing number of initiatives to prevent, mitigate, and improve deficits associated with PICS, practice barriers hinder the delivery of interventions in the community.^8^^,^^9^ Patients at risk of PICS rarely receive post-discharge evaluation from medical and rehabilitation specialists.^10^ Thus, there is a critical need to improve the assessment and implementation of interventions related to PICS.

Impairments of PICS often do not occur in isolation, and patients frequently present with deficits in multiple domains.^11^ Interventions in the intensive care unit (ICU) to reduce the risk of developing PICS are highlighted by the ICU Liberation (A2F) Bundle.^12^^,^^13^ Posthospital medical and rehabilitation care has been promoted as an important component of addressing the long-term sequelae of critical illness survivorship, including quality of life, employment, and mental health, but data on effectiveness remain sparse and mixed.^9^^,^^14^, ^15^, ^16^, ^17^, ^18^, ^19^ Heterogeneity of patient- and treatment-related factors, as well as intervention delivery, may influence outcomes.^14^^,^^16^ Nonetheless, physical rehabilitation after ICU admission is highly recommended in modified Delphi expert initiatives.^20^

The severe acute respiratory syndrome coronavirus 2 pandemic has further highlighted the risk of PICS in patients surviving critical illness caused by coronavirus disease (COVID) of 2019 (COVID-19) and the need for follow-up care to improve outcomes.^21^^,^^22^ Heightened attention in research and clinical practice has emphasized understanding who is at risk for long-term impairments.^23^ Data from several studies demonstrate that physical rehabilitation in the ICU and hospital is associated with patient outcomes at hospital discharge,^24^, ^25^, ^26^ and readmission risk.^27^ However, there is a dearth of evidence on rehabilitation delivery and utilization after hospitalization. Thus, the primary purpose of this study was to describe the participation rates of community-based rehabilitation (physical and occupational therapy) in patients who survived critical COVID-19 and attended an ICU recovery clinic. Second, we analyzed associations between participation in community-based rehabilitation and physical function.

## Methods

### Study design

We conducted a retrospective, observational cohort study with practice analysis among survivors of critical COVID-19 who were seen in an ICU recovery clinic at four academic medical centers in the United States from March 2020 to April 2021 following the first wave of the COVID-19 pandemic. Each of the four medical centers supports a designated ICU recovery clinic providing follow-up care for survivors of critical illness. The study was approved by the medical expedited Internal Review Board at each local academic medical center (University of Kentucky Expedited review No. 47751). Because of the retrospective design of the study, informed consent was waived. Demographic, rehabilitation, and clinical data were extracted and deidentified from the electronic health record. A standardized data dictionary was developed prior to data extraction by the interdisciplinary study personnel. Data were uploaded to a shared REDCap (electronic data capture tool hosted at the University of Kentucky) using data validation techniques to enhance data fidelity across the sites.

### Patient population and setting

We included adult patients (aged ≥18 years) with a primary diagnosis of COVID-19 requiring admission to the ICU because of acute respiratory failure requiring respiratory support, including invasive mechanical ventilation (IMV), bilevel positive airway pressure (BiPAP)/continuous positive airway pressure (CPAP) (noninvasive ventilation), or high-flow nasal cannula (HFNC), and who survived and attended short-term follow-up, ie, 1-3 months after hospital discharge at an ICU recovery clinic at the University of Kentucky, Cleveland Clinic, University of Michigan, or Vanderbilt University Medical Center. We have previously detailed the purpose and patient populations of the ICU recovery clinics.^28^, ^29^, ^30^

### Outcomes

The primary outcome was the percentage distance achieved compared with the predicted distance on the 6-minute walk test (6MWT) at short-term follow-up.^31^ Secondary outcomes at the short-term follow-up included cognition (as measured using the Montreal Cognitive Assessment, or MoCA)^32^ and physical frailty as measured by the Short Physical Performance Battery (SPPB).^33^ Readmission rates at 30 and 90 days were extracted from medical records, and binary outcomes of return to driving and return to work were also quantified for those patients who performed these activities prior to the ICU admission.

### Physical rehabilitation variables

In-hospital rehabilitation was previously examined and published in individuals admitted to the ICU for COVID-19 (supplemental digital content).^26^ The focus of this study was the post-hospital phase of recovery. First, we extracted functional mobility examined with the Activity Measure for Post-Acute Care (AM-PAC) “6-Clicks” (ranged from 6-24; 24=greater functional independence) at or near hospital discharge as an indicator of baseline physical function when leaving the hospital. In addition, the referral status of rehabilitation at hospital discharge was obtained as the percentage of patients referred to inpatient rehabilitation, home health rehabilitation, and ambulatory rehabilitation. We also quantified receipt, ie, actual participation in posthospital community-based rehabilitation, extracted from electronic medical records and/or obtained as a subjective report during patient interviews in the ICU recovery clinic. Specifically, we quantified the participation in community-based rehabilitation, including home health, ambulatory, and specialty physical therapy (PT) and occupational therapy (OT) as binary variables. Because of the small sample of patients receiving OT, we aggregated data to quantify participation in at least 1 community-based rehabilitation session regardless of discipline or location (yes, no).

### Demographic and clinical variables

Demographics and social variables of interest were age, sex, body mass index, race/ethnicity (White/Caucasian, Black/African American, and other), residence location (rural vs urban), distance living from the ICU recovery clinic, and insurance status. Comorbid burden was estimated using the functional comorbidity index. Clinical data from the ICU and hospital stays extracted from the electronic health records included ICU and hospital length of stay (LOS; days); receipt and duration of supplemental oxygen via IMV, BiPAP/CPAP, and HFNC; receipt and duration of extracorporeal support (extracorporeal membrane oxygenation and kidney replacement therapy); tracheostomy (yes, no); receipt of continuous intravenous infusion of a neuromuscular blocker; receipt of vasopressor or inotrope. The Richmond Agitation-Sedation scale score, a surrogate marker of sedative state, was assessed from daily documented nursing notes and quantified by the worst score in the first 72 hours of ICU admission, and diagnosis or indication of delirium in the electronic medical records.

### Statistical analysis

A priori power calculations were not performed because of the design of the study, which used a convenience sample. Continuous data are reported as median and interquartile range (IQR) or mean ± SD, as appropriate. Count data are reported as frequency (n) and percentages (%). Grouped analysis was performed using Kruskal-Wallis because of nonnormality and the chi-square test/Fisher exact test, as appropriate. First, to understand if functional status at discharge influenced rehabilitation participation, we performed univariate comparisons of AM-PAC and participation in at least 1 community-based rehabilitation using the Kruskal-Wallis or chi-square test/Fisher exact test. Mann-Whitney U tests were performed with patients stratified based on participation in at least 1 community rehabilitation session. Multivariate logistic and linear regressions were performed to analyze the association between receipt of posthospital physical rehabilitation and (1) classification of physical frailty on SPPB and (2) performance on 6MWT, respectively, adjusting *a* priori for functional comorbidity index, receipt of IMV, ICU LOS, and days to time of testing (hospital discharge to short-term follow-up) based on clinical expertise and previous research.^23^ Performance on the 6MWT was defined as the percentage of the predicted distance that was achieved. This calculation accounts for age, sex, and body mass index. Consequently, we did not include these variables as indicator variables in the multivariable linear regression. However, these covariates were included in the physical frailty regression analyses. Missing data were not imputed, and complete case analyses were performed. Statistical analyses were performed using SAS 9.4 (SAS Institute, Cary, NC) and IBM SPSS Statistics (Version 29).

## Results

**Fig 1:**
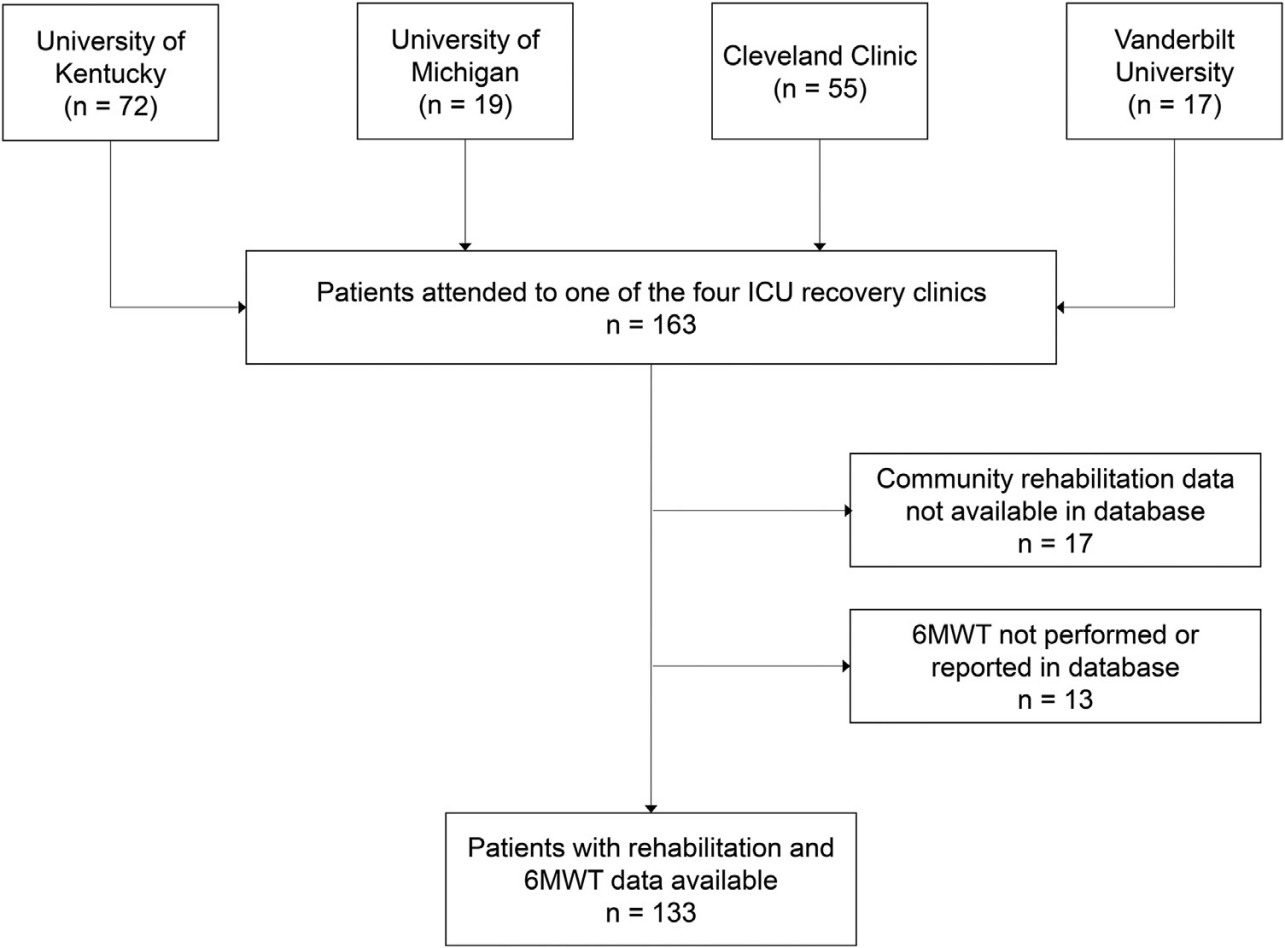
Flow diagram of participating patients.

From March 2020 to April 2021, 163 patients attended one of the four ICU recovery clinics a median of 43 [30-60] days after hospital discharge (figure 1). Patients were a median [IQR] age of 56 [47-64] years, 60% female, and 44% race other races than White/Caucasian (table 1). The median [IQR] ICU and hospital LOS were 14 [8–23] and 22 [15–37] days, respectively. A total of 120 (74%) patients required IMV with a median duration of 12 [7-23] days, 26 individuals required HFNC, and 19 required BiPAP/CPAP. Sixteen individuals (10%) had an unplanned readmission occurring 30 days posthospital discharge. At hospital discharge, the median [IQR] AM-PAC score was 14 [6-19], representing mild-moderate physical function impairments. At discharge, 43 (26%) patients were referred to home health physical rehabilitation, and 51 (31%) patients were referred to an inpatient rehabilitation facility.

**Table 1:** Baseline cohort characteristics

Parameter	Combined Cohort (n=163)	Site 1 (n=72)	Site 2 (n=19)	Site 3 (n=55)	Site 4 (n=17)
Age (y), median [IQR]	56 [47-64]	56 [49-66]	51 [41-60]	58 [49-65]	43 [38-56]
Female, n (%)	97 (59)	45 (63)	11 (58)	34 (62)	7 (41)
Body mass index, kg/m^2^, median [IQR]	33 [28-38]	34 [29-39]	32 [27-37]	30 [28-38]	34 [28-43]
Race/ethnicity					
White/Caucasian, n (%)	92 (56)	42 (58)	8 (42)	31 (56)	11 (65)
Black/African American, n (%)	54 (33)	21 (29)	7 (37)	21 (32)	5 (29)
Other, n (%)	17 (10)	9 (13)	4 (21)	3 (5)	1 (6)
Functional comorbidity index, median [IQR]	3 [1-4]	2 [1-4]	2 [1-3]	3 [2-4]	-
IMV, yes, n (%)	120 (74)	56 (78)	17 (89)	33 (60)	14 (82)
IMV, duration, median [IQR]	12 [7-23]	15 [9-21]	21 [7-59]	8 [3-11]	33 [7-44]
Received tracheostomy, yes, n (%)	40 (25)	20 (28)	9 (47)	3 (5)	8 (47)
RASS, median [IQR]	−2 [−4, −0.1]	−4 [−5, -2]	-2 [-3, 1]	−1 [−2, 0]	-
Received vasopressor, yes, n (%)	86 (53)	45 (63)	15 (79)	16 (29)	10 (59)
Received paralytic, yes, n (%)	71 (44)	34 (47)	10 (53)	19 (35)	8 (47)
Received ECMO, yes, n (%)	19 (12)	10 (14)	4 (21)	0	5 (29)
Received acute kidney support, yes, n (%)	32 (20)	19 (26)	5 (26)	3 (5)	5 (29)
Delirium, yes, n (%)	79 (49)	34 (47)	11 (61)	24 (44)	10 (59)
ICU length of stay (d), median [IQR]	14 [8-23]	16 [10-23]	14 [8-46]	9 [5-18]	34 [8-46]
Hospital length of stay (d), median [IQR]	22 [15-37]	25 [16-37]	22 [11-58]	17 [11-23]	46 [21-56]
Discharge disposition					
Long-term care facility	25 (15)	4 (6)	5 (26)	8 (15)	8 (47)
Inpatient rehabilitation facility	51 (31)	32 (44)	4 (21)	11 (20)	4 (24)
Home with home health	43 (26)	16 (22)	8 (42)	15 (27)	4 (24)
Home without care	44 (27)	20 (28)	2 (11)	21 (38)	1 (6)
AM-PAC at hospital discharge, median [IQR]	14 [6-19]*	8 [6-18]	10 [6-17]	18 [11-20]	-
Time to ICU recovery clinic (d), median [IQR]	43 [30-60]*	33 [26-43]	60 [48-77]	59 [42-85]	-
MoCA, median [IQR]	26 [22-28]*	25 [22-28]	27 [26-28]	25 [21-27]	-
SPPB, median [IQR]	8 [4-11]*	7 [3-10]	9 [6-11]	9 [6-11]	-
6MWD, meters, median [IQR]	282 [150-390]*	180 [88-325]	360 [255-435]	353 [217-429]	372 [253-402]
Percentage 6MWD (%), median [IQR]	44 [27-61]*	30 [16-56]	56 [44-69]	53 [40-75]	-

Abbreviations: ECMO, extracorporeal membrane oxygenation; RASS, Richmond Agitation Sedation Scale.

⁎ AM-PAC, Time to ICU recovery clinic, MoCA, SPPB, 6MWD, and percentage 6MWD were available in 146, 145, 100, 113, 133, and 122 participants, respectively. AM-PAC ranged from 6-24, with higher scores indicating greater functional independence in mobility activities. SPPB ranged from 0-12, with higher scores indicating better physical performance.

### Posthospital rehabilitation

Out of 163, 50 (31%) and 19 (12%) patients reported participating in at least one home health physical and occupational therapy session, respectively. Fifty-one (31%) of 163 patients reported participating in at least one outpatient PT session. No patient reported participating in outpatient occupational therapy. Of the 163, 12 (7%) patients reported receiving both during their recovery, ie, at least 1 home health session and at least 1 outpatient session. In total, 94 out of 163 (58%) patients participated in at least 1 community-based rehabilitation session (either home health or ambulatory); 52 (32%) patients reported not receiving any community rehabilitation, and 17 (10%) patients did not have data available for posthospital rehabilitation.

### Patient-centered outcomes in the ICU recovery clinic

**Fig 2:**
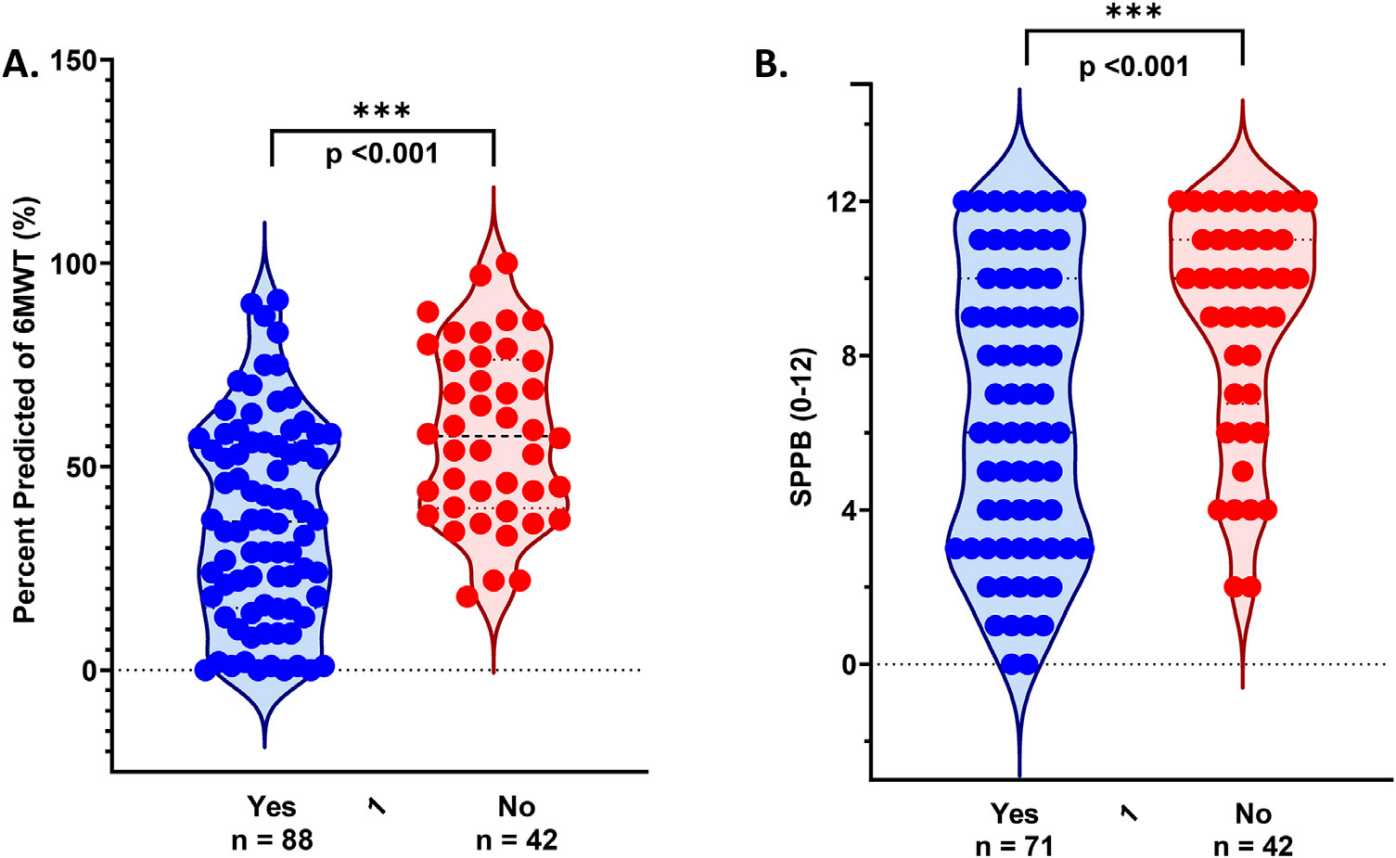
Patient-centered outcomes grouped based on participation in at least 1 community-based rehabilitation session. Patients who participated in at least 1 community rehabilitation session performed worse on the 6MWT (A) and SPPB (B) compared with those not receiving rehabilitation. Mann-Whitney U test performed with *P*<.05. (A) *P*<.001, n=130; at short-term follow-up, 133 patients completed the 6MWT, but n=3 had missing data for community-based rehabilitation not included in this figure. (B) *P*<.001, n=113.

At follow-up in an ICU recovery clinic, 133 out of 163 patients (82%) performed the 6MWT, achieving a median [IQR] of 282 [150-390] meters, equating to 44% [27-61] of the predicted distance. The median SPPB score was 8 out of 12 [4-11] in 113 out of 163 patients completing the test, with 71 (63%) of those patients classified as physically frail (defined as a score ≤9/12). Among 100 individuals completing the MoCA at follow-up, the median score was 26 [22-27], with 37 (37%) classified as having at least mild cognitive impairment (defined as a score ≤24/30). Additionally, 56 out of 126 (44%) patients who were driving before admission had not returned to driving, and 26 (21%) reported driving with new limitations (ie, only short distances in local neighborhoods, only driving during the day, and not driving in rain). Lastly, 13 of 117 (11%) patients working prior to hospitalization reported returning to the same level of occupation at the 3-month time point, while 21 (18%) reported returning to work part-time or with restrictions (ie, reduced capacity). Notably, patients who participated in at least 1 community-based rehabilitation session had longer LOS in the ICU and hospital, worse performance on AM-PAC at hospital discharge, and worse performance on 6MWT and SPPB at short-term follow-up (figures 2A and 2B) compared with individuals not receiving postdischarge rehabilitation.

### Multivariable regression

The variables ICU LOS (odds ratio estimates=1.09, 95% confidence interval: 1.0-1.2, *P*=.007) and participation in at least 1 community-based rehabilitation (odds ratio estimates=2.82, 95% confidence intervalCI: 1.1-7.4, *P*=.036) were significantly associated with the occurrence of physical frailty at short-term follow-up (n=113, table 2). Comorbid burden (β=−0.37, *P*=0.001), ICU LOS (β=−0.004, *P*=.009), time (in days) from hospital discharge testing (β=0.02, *P*=.005), and participation in at least 1 community-based rehabilitation session (β=−10.14, *P*<.001) were significantly associated with the performance on 6MWT (n=121, R^2^=0.31, F=10.3, *P*<.001; table 2).

**Table 2:** Multivariable regression of primary dependent outcomes

Logistic Regression Predicting Physical Frailty (n=113)					
Indicator Variable	Odds Ratio	95% CI		*P*=.05	
Age (y)	1.02	0.98, 1.07		0.389	
Females compared with males	0.62	0.21, 1.84		0.384	
BMI (kg/m^2^)	1.06	0.99, 1.14		0.111	
Functional comorbidity index	1.28	0.92, 1.77		0.143	
Required IMV compared with HFNC/BiPAP	0.33	0.10, 1.11		0.072	
ICU length of stay (d)	1.09	1.03, 1.17		0.007	
Days from discharge to ICU recovery clinic testing	1.012	0.99, 1.04		0.384	
Received community-based rehabilitation after discharge, yes	2.82	1.07, 7.41		0.036	

Linear Regression Predicting Percent Predicted of 6MWD (n=120)					
Indicator Variable	B	S.E.	t		*P*=.05

Intercept	64.9	5.85	11.09		<.0001
Functional comorbidity index	−3.74	1.06	−3.53		.0006
Required IMV compared with HFNC/BiPAP	−5.10	4.61	−1.11		.2701
ICU length of stay (d)	−0.38	0.14	−2.64		.0094
Days from discharge to ICU recovery clinic testing	0.21	0.07	2.88		.0047
Received community-based rehabilitation after discharge, yes	−13.9	4.11	−3.40		.0009

Abbreviations: BMI, body mass index.

## Discussion

In this observational study of survivors of critical COVID-19 who attended an ICU recovery clinic about 1.5 months after discharge, we found that the occurrence of physical and cognitive impairment was high, and about two-thirds participated in community-based physical or occupational rehabilitation. Notably, participation in at least 1 community-based rehabilitation was associated with longer LOS in the ICU and hospital and worse performance on the 6MWT. Thus, the findings suggest that patients with the greatest need for rehabilitation are getting referred and participating in community-based rehabilitation. However, over 1 in 3 ICU survivors of COVID never received physical or occupational therapy in the home or community after hospital discharge.

Data on ICU survivors prior to the COVID-19 pandemic have demonstrated very low rates and frequencies of posthospital home health rehabilitation, with the majority receiving a single home health session.^8^^,^^34^ In addition, approximately 1 in 3 Medicare beneficiaries surviving a hospitalization who are discharged with a home health referral actually do not receive home health care.^35^ Our findings confirm the findings of these prior studies and build upon them by demonstrating that patients who survive critical illness due to critical COVID-19 are at high risk of physical and cognitive deficits related to PICS, yet 1 in 3 never received community-based rehabilitation, highlighting the need for improvement in care pathways following hospitalization for critical illness. Interestingly, we found that receipt of posthospital rehabilitation was associated with worse 6MWD and physical frailty at follow-up in an ICU recovery clinic. This finding probably reflects the fact that survivors at hospital discharge with worse functional status were more likely to be referred for community-based rehabilitation services, to attend a post-ICU clinic visit, and to have a worse functional status at short-term follow-up. It also may suggest that the dose of community-based rehabilitation early in the postdischarge phase may not routinely be adequate to reliably improve functional status. This result emphasizes the need for future studies that report and standardize the intensity of rehabilitation after hospital discharge to better understand the role of rehabilitation in this vulnerable period after hospitalization.

We also demonstrated that 37% of our survivors met the criteria for mild cognitive impairment on MoCA, which is consistent with findings in ICU survivors with COVID-19^36^ and non-COVID-19 populations.^6^ Multidomain rehabilitation services, including physical, occupational, and speech therapy interventions that can target cognitive function, are likely needed to address the deficits that occur following critical illness, yet they are not routinely provided to ICU survivors as recommended by experts in Delphi consensus statements.^14^^,^^37^ To emphasize, according to our data, only 12% of patients participated in home health occupational therapy, and no patient reported receiving OT in an ambulatory setting. We did not quantify outpatient speech therapy in this study, but the findings are important to note in the future. Our data, along with previous studies, demonstrated that further research is necessary to understand why patients are not receiving community-based rehabilitation, despite the high rates of physical and cognitive impairments related to PICS.

Physical, cognitive, and mental functioning are imperative to a full recovery after ICU admission, and persistent deficits in these domains can lead to difficulty in returning to activities of daily living, including driving. Driving is imperative for independence in multiple areas of the USA. Previous studies have shown that 13%, 68%, 77%, and 84% of survivors following critical illness resume driving by 1, 3, 6, and 12 months after hospital discharge,^38^^,^^39^ suggesting delayed resumption of driving related to mental, cognitive, physical, and supportive care needs in the year following a critical illness.^38^ In our study, 44% of patients who were driving before admission had not returned to driving ∼2 months after hospital discharge, with an additional 21% reporting driving with new limitations. Clinically, posthospital rehabilitation, as well as vocational rehabilitation programs, may increase the likelihood of returning to driving as well as other activities of daily living. However, more research is necessary to understand the natural recovery trajectories with enhanced descriptors of the patient and rehabilitation factors that facilitate or hinder recovery.

### Strengths and limitations

Moderate rates of rehabilitation postdischarge may be reflected by this very sick patient population. It should be emphasized that this sample has a high risk of representation and survivor bias, because patients who attended the clinic may be more likely to have the financial resources and insurance coverage for community-based rehabilitation. We could not quantify the frequency or dosage of posthospital rehabilitation because of difficulty with patient recall and significant disconnection of the electronic health record in the USA. It is very likely that some patients only received a single physical rehabilitation session, whereas others may have participated in rehabilitation at regular intervals throughout their recovery. The retrospective design of our study limits the ability to draw causal inference from our analyses and increases the risk of selection bias. The study is at risk of selection bias in the sampling frame (cross-sectional and early recovery) because we only included patients recovering from COVID who voluntarily attended ICU recovery clinics. Moreover, the study is at high risk of recall bias that may compromise the validity of the results. Thus, it does not account for patient-specific factors that may also impact recovery, including transportation, social and financial support, understanding of post-ICU deficits, and distance to the clinic. Lastly, like all observational studies, there is likely residual confounding that is not accounted for despite adjusting for important covariates in our models.

Our study had several strengths. We enrolled a representative sample of ICU survivors from across 4 academic medical centers, increasing generalizability, with the use of relevant clinical physical performance measures. However, our sample should be interpreted with caution, because we did not capture the individuals who did not attend or who were never referred to the ICU recovery clinic. Moreover, roughly 10% of our sample was missing, leading to attrition bias. Additionally, we collected detailed data on functional outcomes within the first few months after hospital discharge, an understudied and vulnerable period of recovery after critical illness.

## Conclusions

In this observational study of survivors of critical illness from COVID-19 who attended an ICU recovery clinic, we found that, despite a high risk of physical and cognitive deficits related to PICS, one-third of survivors never participated in a community-based rehabilitation program after discharge from the hospital. Future studies should investigate the patient, clinical, and social factors that influence trajectories of recovery after critical COVID.

## Supplier

a. REDCap (Research Electronic Data Capture).
b. SAS 9.4 (SAS Institute, Cary, NC)
c. IBM SPSS Statistics (Version 29)

## Disclosure

The authors have nothing to disclose.

## Appendix Supplementary materials

Image, application 1

## Footnotes

Supplementary material associated with this article can be found, in the online version, at doi:10.1016/j.arrct.2025.100484.
