# Supplementary material for: Community-Based Rehabilitation and Patient-Centered Outcomes in Survivors of Critical COVID-19 Attending an Intensive Care Recovery Clinic
Source: Arch Rehabil Res Clin Transl. 2025 Jun 15;7(3):100484. doi: 10.1016/j.arrct.2025.100484 (PMC12447205; doi:10.1016/j.arrct.2025.100484)
Supplement: Supplementary file 1 [file mmc1.docx]

**Supplemental Digital Content**

For this cohort, we extracted data from previously published study on the hospital-based rehabilitation including received physical therapy treatment (yes, no), received occupational therapy treatment (yes, no); time to first physical therapy and occupational therapy session (days from admission to participation in the initial session); frequency of physical and occupational therapy during hospitalization as a proportion of sessions completed per hospital days (percentage) and proportion of sessions completed per number of days from completion of the evaluation to discharge (percentage); percentage of session completed as a co-treatment (physical and occupational therapy performed concurrently); and average duration of rehabilitation sessions in minutes. We used a surrogate marker of type of rehabilitation defined as the highest level of mobility (Johns Hopkins-Highest Level of Mobility scale [JH-HLM]) achieved with the first three and the last rehabilitation session; we calculate the mean across the sessions and the change from the first to the final session. Utilizing the frequency of rehabilitation in the hospital and the surrogate marker of the type of rehabilitation, we calculated a proxy marker of dosage (average JH-HLM multiplied by the frequency of physical rehabilitation).

**In-Hospital Rehabilitation**: The mean time to the initial physical therapy and occupational therapy sessions were 10 ± 9.8 days and 11 ± 10.5 days after hospital admission, respectively. Patients received physical therapy treatment sessions a mean 21 ± 13% of hospital days with average duration of 32 ± 9.1 minutes per session. Patients received occupational therapy treatment sessions a mean 18 ± 13% of hospital days with average duration of 35 ± 13 minutes per session. Physical and occupational therapy were delivered as co-treatment 52 ± 42% of all sessions. The proportions of days receiving either physical or occupational therapy was a mean 31 ± 19% for entire hospitalization and 79 ± 78% when only examining the time from the initial rehabilitation evaluation to hospital discharge, both adjusted for co-treatment. The initial and final recorded JH-HLM scores were 3.8 ± 2.0 and 4.9 ± 2.0, respectively. At initial physical therapy evaluation, the AM-PAC IMSF was mean 13.7 ± 6.9 that improved to 18.8 ± 7.1 at hospital discharge.

**Supplemental Table.** Physical rehabilitation parameters delivered in the hospital.

| **Parameter** | **Combined Cohort**  **(n =163)** | **Kentucky**  **(n = 72)** | **Michigan**  **(n = 19)** | **Cleveland Clinic**  **(n = 55)** | **Vanderbilt**  **(n = 17)** | **ANOVA**  **(p=0.05)** |
| --- | --- | --- | --- | --- | --- | --- |
| Time to initial PT, days, mean (SD) | 10.1 ± 9.8 | 7.3 ± 7.1 | 10.8 ± 14.1 | 10.7 ± 6.4 | 19.8 ± 15.5 | p < 0.001 |
| Time to initial OT, days, mean (SD) | 10.9 ± 10.5 | 7.7 ± 7.1 | 18.7 ± 16.5 | 10.1 ± 6.5 | 19.9 ± 15.4 | p < 0.001 |
| Number of PT sessions | 6.5 ± 5.8 | 6.3 ± 4.5 | 11.4 ± 8.8 | 4.2 ± 4.2 | 9.1 ± 7.1 | p < 0.001 |
| Number of OT sessions | 5.1 ± 4.1 | 5.7 ± 3.9 | 4.9 ± 5.1 | 3.5 ± 3.5 | 7.0 ± 4.4 | p = 0.008 |
| Duration of PT, minutes, mean (SD) | 31.9 ± 9.1 | 28.4 ± 3.5 | 39.5 ± 9.7 | 33.6 ± 11.6 | - | p < 0.001 |
| Co-treatment, percentage, mean (SD) | 3.1 ± 3.3 | 4.9 ± 3.1 | 1.6 ± 2.3 | 0.25 ± 0.5 | - | p < 0.001 |
| Frequency of PT in hospital, %, mean (SD) | 23 ± 11 | 22 ± 10 | 33 ± 15 | 21 ± 12 | 21 ± 10 | p < 0.001 |
| Frequency of PT post eval, %, mean (SD) | 46 ± 31 | 36 ± 31 | 44 ± 18 | 64 ± 30 | 35 ± 12 | p < 0.001 |
| AM-PAC at initial eval, mean (SD) | 13.3 ± 6.3 | 11.4 ± 6.3 | 11.9 ± 5.9 | 16.1 ± 5.6 | - | p < 0.001 |
| Time to sitting EOB | 14.0 ± 11.2 | 15.0 ± 12.4 | 17.6 ± 15.0 | 11.4 ± 7.0 | - | p = 0.406 |
| Dosage (JHLHM levels*Frequency) | 1.3 ± 1.0 | 0.98 ± 0.7 | 1.5 ± 0.9 | 1.6 ± 1.3 | - | p = 0.002 |
